# Comparative binding properties of the tau PET tracers THK5117, THK5351, PBB3, and T807 in postmortem Alzheimer brains

**DOI:** 10.1186/s13195-017-0325-z

**Published:** 2017-12-11

**Authors:** Laetitia Lemoine, Per-Göran Gillberg, Marie Svedberg, Vladimir Stepanov, Zhisheng Jia, Jinghai Huang, Sangram Nag, He Tian, Bernardino Ghetti, Nobuyuki Okamura, Makoto Higuchi, Christer Halldin, Agneta Nordberg

**Affiliations:** 1 Division of Translational Alzheimer Neurobiology, Department of Neurobiology, Care Sciences and Society, Karolinska Institutet, Stockholm, Sweden; 2 Department of Clinical Neuroscience, Center for Psychiatric Research, Karolinska Institutet, Stockholm, Sweden; 3 Institute of Fine Chemicals, East China University of Science and Technology, Shanghai, China; 4 Department of Pathology & Laboratory Medicine, Indiana University School of Medicine, Indianapolis, IN USA; 5 Division of Pharmacology, Faculty of Medicine, Tohoku Medical and Pharmaceutical University, Tohoku, Japan; 6 National Institute of Radiological Sciences, National Institutes for Quantum and Radiological Science and Technology, Chiba, Japan; 7 Department of Geriatric Medicine, Karolinska University Hospital, Huddinge, Sweden

**Keywords:** Alzheimer’s disease, THK5117, ^11^C-THK5351, T807, AV1451, PBB3, Tau imaging, autoradiography, Tau PET, Tau PET tracers, Deprenyl

## Abstract

**Background:**

The aim of this study was to compare the binding properties of several tau positron emission tomography tracers—THK5117, THK5351, T807 (also known as AV1451; flortaucipir), and PBB3—head to head in the same human brain tissue.

**Methods:**

Binding assays were performed to compare the regional distribution of ^3^H-THK5117 and ^3^H-THK5351 in postmortem tissue from three Alzheimer’s disease (AD) cases and three control subjects in frontal and temporal cortices as well as in the hippocampus. Competition binding assays between THK5351, THK5117, PBB3, and T807, as well as off-target binding of THK5117 and T807 toward monoamine oxidase B (MAO-B), were performed using binding assays in brain homogenates and autoradiography of three AD cases.

**Results:**

Regional binding of ^3^H-THK5117 and ^3^H-THK5351 was similar, except in the temporal cortex, which showed higher ^3^H-THK5117 binding. Saturation studies demonstrated two binding sites for ^3^H-THK5351 (*K*_d1_ = 5.6 nM, B_max_ = 76 pmol/g; *K*_d2_ = 1 nM, B_max_ = 40 pmol/g). Competition studies in the hippocampus between ^3^H-THK5351 and unlabeled THK5351, THK5117, and T807 revealed super-high-affinity sites for all three tracers (THK5351 *K*_i_ = 0.1 pM; THK5117 *K*_i_ = 0.3 pM; T807 *K*_i_ = 0.2 pM) and an additional high-affinity site (THK5351 *K*_i_ = 16 nM; THK5117 *K*_i_ = 20 nM; T807 *K*_i_ = 78nM). ^18^F-T807, ^11^C-THK5351, and ^11^C-PBB3 autoradiography of large frozen sections from three AD brains showed similar regional binding for the three tracers, with lower binding intensity for ^11^C-PBB3. Unlabeled THK5351 and T807 displaced ^11^C-THK5351 to a similar extent and a lower extent, respectively, compared with ^11^C-PBB3. Competition with the MAO-B inhibitor ^3^H-l-deprenyl was observed for THK5117 and T807 in the hippocampus (THK5117 *K*_i_ = 286 nM; T807 *K*_i_ = 227 nM) and the putamen (THK5117 *K*_i_ = 148 nM; T807 *K*_i_ = 135 nM). ^3^H-THK5351 binding was displaced using autoradiography competition with unlabeled THK5351 and T807 in cortical areas by 70–80% and 60–77%, respectively, in the basal ganglia, whereas unlabeled deprenyl displaced ^3^H-THK5351 binding by 40% in the frontal cortex and 50% in the basal ganglia.

**Conclusions:**

THK5351, THK5117, and T807 seem to target similar binding sites, but with different affinities, whereas PBB3 seems to target its own binding site. Both THK5117 and T807 demonstrated off-target binding in the hippocampus and putamen with a ten times lower binding affinity to the MAO-B inhibitor deprenyl compared with ^3^H-THK5351.

**Electronic supplementary material:**

The online version of this article (doi:10.1186/s13195-017-0325-z) contains supplementary material, which is available to authorized users.

## Background

Accumulation of tau protein is one of the main hallmarks of Alzheimer’s disease (AD), along with amyloid deposition and astrocytosis. In recent years, several attempts have been made to develop positron emission tomography (PET) tracers that are able to visualize tau deposits in vivo.

The complexity of developing a ligand targeting tau protein is increased because of its intracellular location. The first compound used to target tau deposits in vivo was 2-(1-{6-[(2-[fluorine-18]fluoroethyl)(methyl)amino]-2-naphthyl}-ethylidene)malononitrile (FDDNP), which was developed as an amyloid tracer but also showed some binding to neurofibrillary tangles (NFTs). Unfortunately, because of its affinity for amyloid plaques and lack of selectivity to NFTs, FDDNP is not suitable for tau imaging [1–3]. Recently, several tau PET tracers have been developed and tested in vitro and in preclinical imaging, showing good results and leading to their inclusion in clinical studies. In this study, we focused on the THK tracer family as well as PBB3 and T807. First, ^11^C-PBB3 has been reported to be a good candidate for in vivo PET imaging and autoradiography with ^11^C-PBB3 in PS19-transgenic mice, showing binding in the same area as the fluorescent Congo red derivative (*trans*,*trans*)-1-fluoro-2,5-bis(3-hydroxycarbonyl-4-hydroxy)styrylbenzene, indicating the presence of tau filaments [4, 5]. Moreover, autoradiography experiments in human brain tissue showed binding to NFTs in the hippocampus and no colocalization with ^11^C-Pittsburgh compound B (PIB) [4]. In vitro characterization of AV-1451 using autoradiography showed a good affinity for paired helical filaments (PHF-tau), as well as good colocalization, in comparison with PHF-tau immunohistochemistry, in addition to a correlation with the NFT deposition in the different Braak stages [6–9]. Binding assay experiments performed on AD brain homogenates using ^3^H-THK5117 demonstrated several binding sites in both temporal and hippocampal regions [10]. THK5117 and THK5351, the latest derivatives of the aryquinoline series, have shown good affinity for PHF-tau [11]. THK5351, which is the *S*-form of THK5151, has been reported to bind less than other tau tracers to white matter [12, 13]. Because extensive in vitro data are available for all the tracers in AD brain tissue, some studies have been focused on non-AD tauopathies [14]. In recent studies, both in vitro and in vivo experiments have shown that the different tau PET tracers bind to different tau deposits (*see* [15] for review). The tau protein can have either three repeats (3R) or four repeats (4R) of the microtubule-binding domain regarding inclusion or exclusion of exon 10 during alternative splicing. The affinity of the tracer appears to depend on the inclusion of 3R, 4R, or both [16–19].

Off-target binding of the different tau tracers has been observed in regions poor in PHF-tau. The location of this off-target binding in the basal ganglia led some research groups to suggest the possibility of binding to monoamine oxidase (MAO) (*see* [15] for review). In a recent study done at McGill University, Ng et al. showed that pretreatment with selegiline, a MAO-B inhibitor, reduced the in vivo PET uptake of ^18^F-THK5351 [20] and confirmed those results in vitro. Furthermore, our group recently showed that the affinity between MAO-B and THK5117 occurred at around 300 nM, implying that it should not affect the signal observed by PET [21]. Previous in vitro studies have also indicated that T807 binds to MAO-A [22, 23].

The aim of this study was to compare the two THK compounds THK5117 and THK5351 with T807 and PBB3 in head-to-head autoradiography and binding assay studies in the same human brain tissue. To our knowledge, this is the first report of the binding of ^11^C-THK5351 using autoradiography of large brain sections.

## Methods

### Chemicals

1-Fluoro-3-((2-(4-([^3^H]methylamino)phenyl)quinolin-6-yl)oxy)propan-2-ol (^3^H-THK5117; specific activity 2.2 GBq/μmol) and unlabeled THK5117 were custom-synthesized by Quotient Bioresearch/Pharmaron (Cardiff, UK). ^3^H-THK5351, ^11^C-THK5351 [(*S*)-1-fluoro-3-(2-(6-([^11^C]methylamino)pyridin-3-yl)quinolin-6-yloxy)propan-2-ol], ^11^C-PBB3 [(5-((1*E*,3*E*)-4-(6-[^11^C] methylamino)pyridin-3-yl)buta-1,3-dien-1-yl)benzo[*d*]thiazol-6-ol], and ^18^F-T807 [7-(6-[^18^F]fluoropyridin-3yl)-5*H*-pyrido(4,3-b)indole] were synthesized and labeled at the Centre for Psychiatric Research in the Department of Clinical Neuroscience (Karolinska Institutet, Solna, Sweden). NO and MH provided the THK5351 and PBB3 precursors, respectively. Unlabeled T807 and *tert*-butyloxycarbonyl-protected precursors were synthesized by HT. (*R*)-(−)-deprenyl hydrochloride was purchased from Tocris Bioscience (Bristol, UK).

### In vitro binding assay

Postmortem brain tissues from three patients with AD and three healthy control subjects were used for the in vitro binding assays. All the brain tissue, which came from the Netherlands Brain Bank, had been homogenized in PBS containing a protease/phosphatase inhibitor (*see* Table 1).

**Table 1:** Demographic data

		Sex (M/F)	Age (years)	Braak stage	ApoE	Postmortem delay (h:minutes)
Regional distribution comparison	AD	M	78	5	4/4	6:35
		F	75	5	4/4	5:50
		F	81	5	4/3	6:15
	Control	F	77	1	3/3	2:55
		F	84	1	3/3	6:55
		M	81	2	3/3	7:55
^3^H-THK5351 competition study	AD	M	77	6	NA	6:35
		F	86	6	NA	4:20
		F	75	5	4/4	5:50
^3^H-THK5351 saturation study	AD	F	75	5	4/4	5:50
Large frozen section autoradiography	AD1	N/A	59	N/A	3/3	4:20
	AD2	N/A	73	N/A	3/3	1:45
	AD3	N/A	59	N/A	3/4	10:45

*Abbreviations: AD* Alzheimer’s disease, *ApoE* Apolipoprotein E, *N/A* Not applicable

Summary of the demographic data for the postmortem brain samples used in the experiments

The saturation binding experiment was carried out using increasing concentrations of ^3^H-THK5351 (0.1–250 nM) in hippocampus AD brain homogenate (0.2 mg/ml) to determine the dissociation constant (*K*_d_). Nonspecific binding was determined using 1 μM unlabeled THK5117. After 2-h incubation at room temperature, the binding assay was terminated by filtration through glass fiber filters presoaked for at least 3 h in 0.3% polyethylenimine. To do so, the filters were rinsed and filtered three times using cold binding buffer, and then the radiation on the filter was quantified using a scintillation counter (Beckman Coulter, Brea, CA, USA).

The regional binding distribution comparison between ^3^H-THK5351 and ^3^H-THK5117 was carried out in postmortem frontal and temporal cortical and hippocampal tissue from the brains of three patients with AD and three control subjects. The protocol was similar for ^3^H-THK5351 (1.5 nM) and ^3^H-THK5117 (3 nM): 0.1 mg of tissue in PBS + 0.1% bovine serum albumin (BSA) to a final volume of 500 μl, followed by incubation for 2 h at room temperature. The nonspecific binding was determined using 1 μM unlabeled THK5351 or unlabeled THK5117. After 2-h incubation at room temperature, the competition binding assay was stopped using filtration through glass fiber filters, and the radiation was then quantified using the scintillation counter.

Competition binding studies using postmortem hippocampus brain homogenate (0.2 mg/ml tissue) from three patients with AD were performed using ^3^H-THK5351 (1.5 nM) as well as increasing concentrations of unlabeled THK5351 (10^−14^ to 10^−5^ nM), THK5117 (10^−14^ to 10^−5^ nM), and T807 (10^−14^ to 10^−5^ nM), to determine the inhibition constant (*K*_i_). After 2-h incubation at room temperature, the binding assay was terminated by filtration through glass fiber filters presoaked for at least 3 h in 0.3% polyethylenimine. To do so, the filters were rinsed and filtered three times using cold binding buffer, and then the radiation on the filter was quantified using a Beckman Coulter scintillation counter. The data for the regional binding distribution studies were analyzed using Prism version 7.0 software for Mac (GraphPad Software Inc., La Jolla, CA, USA), and two-way analysis of variance with multiple comparisons was performed.

Competition binding studies using postmortem brain homogenates from the hippocampus and putamen (0.2 mg/ml tissue) of two patients with AD were performed using ^3^H-deprenyl (10 nM in Na-K phosphate buffer, pH 7.4) and increasing concentrations of unlabeled THK5117 (10^−14^ to 10^−5^ M) and T807 (10^−14^ to 10^−5^ M) to determine the off-target binding of the tau PET tracers. After 2-h incubation at room temperature, the binding assay was terminated by filtration through glass fiber filters presoaked for at least 3 h in 0.3% polyethylenimine. To do so, the filters were rinsed and filtered three times using cold binding buffer, and then the radiation on the filter was quantified using a Beckman Coulter scintillation counter. The data from all the binding studies were analyzed using Prism version 7.0 software.

### In vitro autoradiography experiment

Postmortem frozen left brain hemispheres from three patients with AD were obtained from the Neuropathology of Dementia Laboratory (Indiana University School of Medicine, Indianapolis, IN, USA) and used for the autoradiography experiment. The frozen sections were allowed to reach room temperature, preincubated for 10 minutes with PBS + 0.1% BSA (pH 7.4), and then incubated for 1 h at room temperature with ^3^H-THK5351 (3 nM) or ^3^H-THK5117 (3 nM). The sections were rinsed three times in cold buffer for 5 minutes, followed by a quick dip in cold distilled water. Nonspecific binding was determined using 10 μM unlabeled THK5351 or 10 μM unlabeled THK5117. After waiting 24 h for the sections to dry, the sections were apposed to a tritium standard on a phosphoplate for 3 days and then scanned using a BAS-2500 phosphor imager (Fujifilm, Tokyo, Japan).

Six adjacent sections from AD1 (*see* Table 1) were used for competition autoradiography of unlabeled THK5351, T807, and deprenyl. After reaching room temperature, the frozen sections were preincubated for 10 minutes with PBS (pH 7.4) and then incubated for 1 h at room temperature with ^3^H-THK5351 (1.5 nM) in addition to 10 μM unlabeled THK5351; 10 μM unlabeled deprenyl; 10 μM unlabeled T807; 10 μM unlabeled THK5351 and 10 μM unlabeled deprenyl; 10 μM unlabeled T807 and 10 μM unlabeled deprenyl. The sections were rinsed three times in cold buffer for 5 minutes, followed by a quick dip in cold distilled water. Nonspecific binding was determined using 10 μM unlabeled THK5351 or 10 μM unlabeled THK5117. After waiting 24 h for the sections to dry, the sections were apposed to a tritium standard on a phosphor plate for 3 days and then scanned using the BAS-2500 phosphor imager.

Adjacent sections were used for autoradiography with ^11^C-THK5351, ^11^C-PBB3, and ^18^F-T807. The frozen sections were allowed to reach room temperature, incubated for 30 minutes at room temperature with ^11^C-THK5351 (0.4–0.56 nM; specific activity 356–532 GBq/μmol) or ^11^C-PBB3 (0.25–0.34 nM; specific activity 442–794 GBq/μmol) or for 55 minutes at room temperature with ^18^F-T807 (0.04 nM; specific activity 281 GBq/μmol). We used 10 μM unlabeled THK5351, 10 μM unlabeled PBB3, and 10 μM unlabeled T807 to determine the extent of nonspecific binding for each tracer. The sections were rinsed three times for 5 minutes each with cold binding buffer, followed by a quick dip in cold distilled water. The sections were dried and exposed for 1 h (for ^11^C-THK5351 and ^11^C-PBB3) or for 3 h (^18^ F-T807) on a phosphor imaging plate and then read using the BAS-2500 phosphor imager.

For all the autoradiography studies, the regions of interest were drawn manually on the autoradiogram using multigauge software and were used for the semiquantitative analyses. Photostimulated luminescence per square millimeter (PSL/mm^2^) was transformed to picomoles per cubic millimeter using a transformation factor that took into account the resolution of the image, the pixel value for the concentration of ligand, and the thickness of the sections: (PSL/mm^2^ value)/([pixel value per pmol] × [(resolution/1000)^2^] × [cryosection thickness]/1000).

## Results

**Fig. 1:**
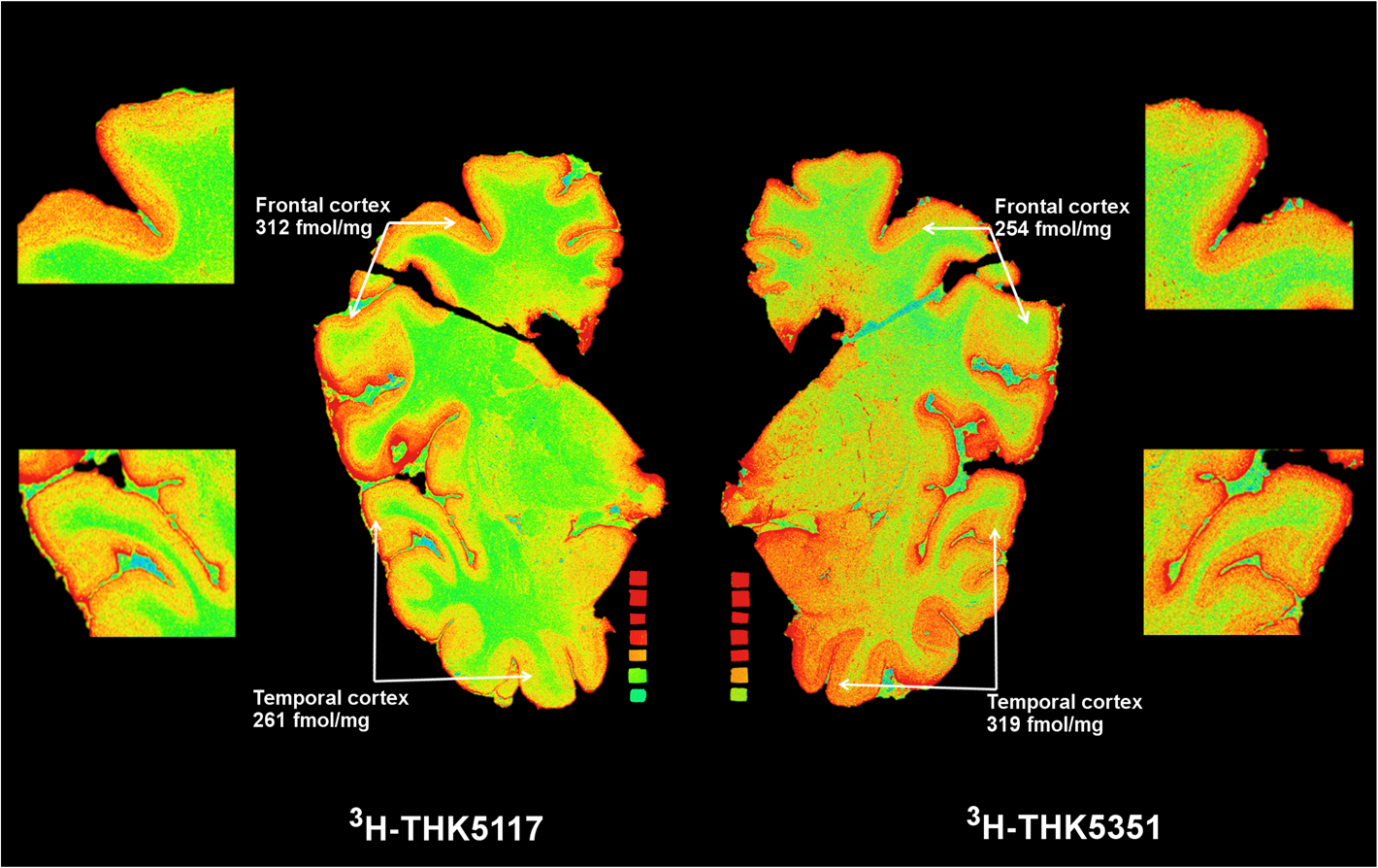
In vitro comparison ^3^H-THK5351 and ^3^H-THK5117 binding. Autoradiography comparison of total binding of ^3^H-THK5117 and ^3^H-THK5351 on large frozen postmortem brain sections. *Red* = highest binding; *blue* = lowest

Autoradiography studies of ^3^H-THK5117 and ^3^H-THK5351 showed similar regional distribution in both frontal and temporal cortex areas. An example of the autoradiography results for AD1 tissue is presented in Fig. 1.

### Regional distribution comparison between THK5351 and THK5117 using binding assays and autoradiography

**Fig. 2:**
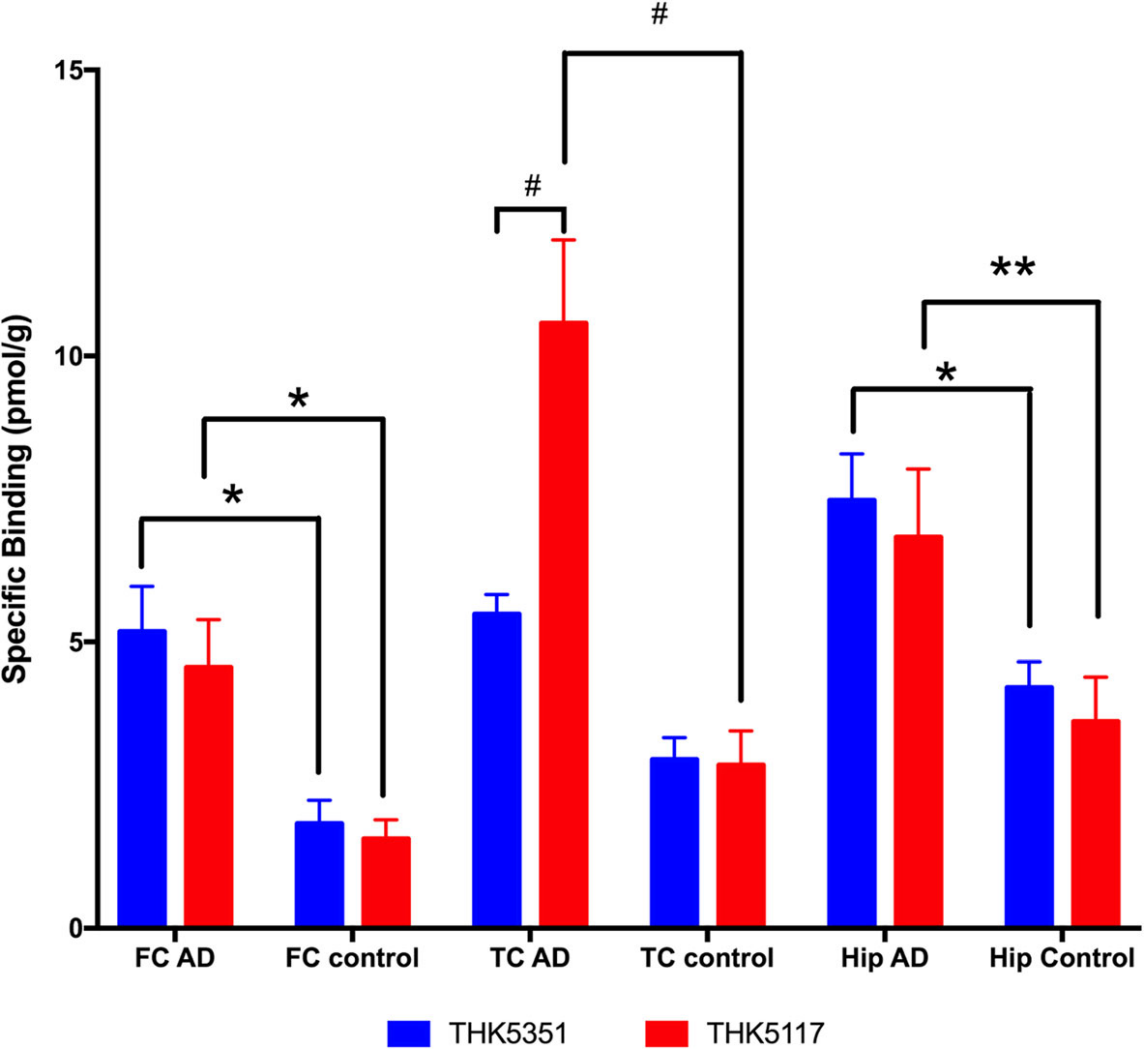
Regional binding distribution in brain homogenate. Regional binding distribution of ^3^H-THK5117 and ^3^H-THK5351 in postmortem frontal cortex, temporal cortex, and hippocampus tissue from three patients with Alzheimer’s disease and three control subjects. Error bars represent the SEM from three experiments in triplicate. Two-way analysis of variance multiple comparisons with Prism software was performed. * *p* < 0.05, ** *p* < 0.005, ^#^ *p* < 0.0001. *AD* Alzheimer’s disease, *FC* Frontal cortex, *Hip* Hippocampus, *TC* Temporal cortex

The regional binding distributions of ^3^H-THK5351 and ^3^H-THK5117 are shown in Fig. 2 (*see* Table 1 for demographic data). The regions studied included the frontal cortex, the temporal cortex, and the hippocampus. The regional binding distribution of the two THK compounds was similar in both AD and control tissue (Fig. 2). The only significant difference between ^3^H-THK5351 and ^3^H-THK5117 binding was in the temporal cortex, with more extensive binding of ^3^H-THK5117 than of ^3^H-THK5351 (*p* < 0.0001) (Fig. 2). For all three regions, the extent of binding in control tissue was significantly lower than in AD tissue.

### Saturation curve for ^3^H-THK5351

**Fig. 3:**
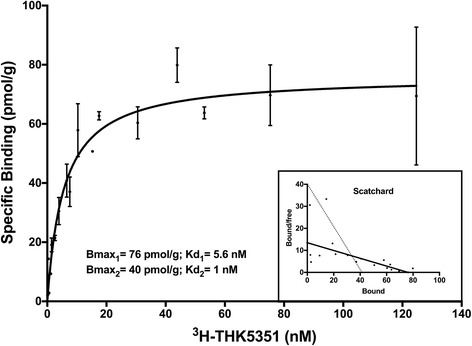
Saturation binding assay. Saturation binding curve in hippocampus brain homogenate using increasing concentrations of ^3^H-THK5351 (0.1–150 nM) and the corresponding Scatchard plot. *K*_d_ Dissociation constant

The saturation binding assay results from increasing concentrations of ^3^H-THK5351 in hippocampus AD brain homogenate are presented in Fig. 3. Saturation occurred with a B_max_ of 76 ± 4.07 pmol/g and a *K*_d_ of 5.3 ± 1.16 nM. An additional binding site was observed on the Scatchard plot. The dotted line in Fig. 3, drawn manually, shows a B_max_ of 40 pmol/mg and a *K*_d_ of 1 nM.

### Competition binding study comparison between ^3^H-THK5351 and unlabeled THK5351, THK5117, and T807

**Fig. 4:**
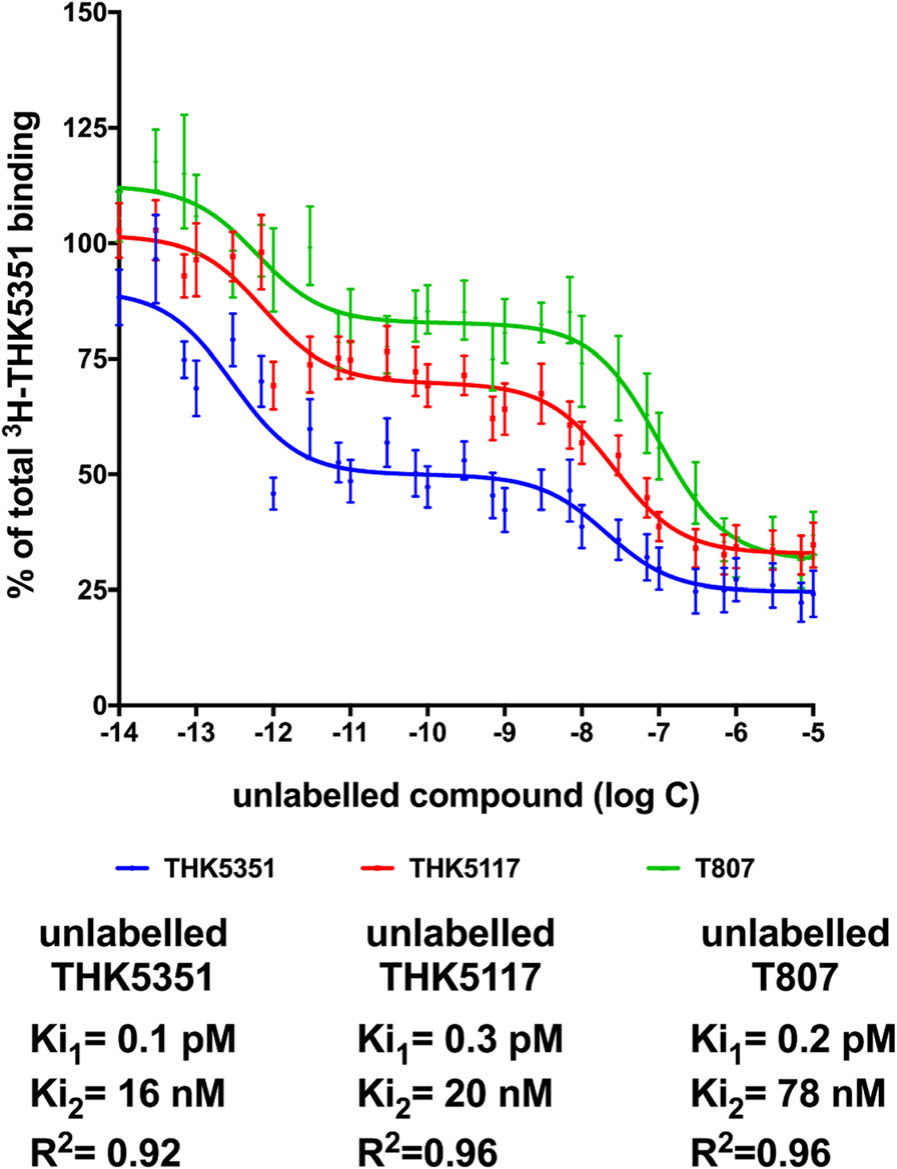
Competition binding assay with ^3^H-THK5351 and unlabeled THK5351, THK5117, and T807. Competition binding studies using ^3^H-THK5351 (1.5 nM) binding in postmortem hippocampus brain homogenates from three patients with Alzheimer’s disease using increasing concentrations of unlabeled THK5351, unlabeled THK5117, or unlabeled T807 (1.10^−14^ to 1.10^−5^ M). Error bars represent the SEM from three experiments in triplicates using two tissue samples for unlabeled THK5351 and THK5117 and three tissue samples for unlabeled T807. *K*_i_ Inhibition constant

The results of the competition binding study using ^3^H-THK5351 and increasing concentrations of unlabeled THK5351, THK5117, and T807 are presented in Fig. 4. We observed more than one binding site for the three unlabeled compounds. THK5117, THK5351, and T807 behaved similarly, competing with ^3^H-THK5351 at low concentrations. The *K*_i1_ values were 0.1 pM, 0.3 pM, and 0.2 pM for THK5351, THK5117, and T807, respectively (Fig. 4). The *K*_i2_ values were 16 nM for THK5351 and 20 nM for THK5117. The second competing site for T807 had a *K*_i2_ value of 78 nM.

### Autoradiography comparison and competition between three tau tracers

**Fig. 5:**
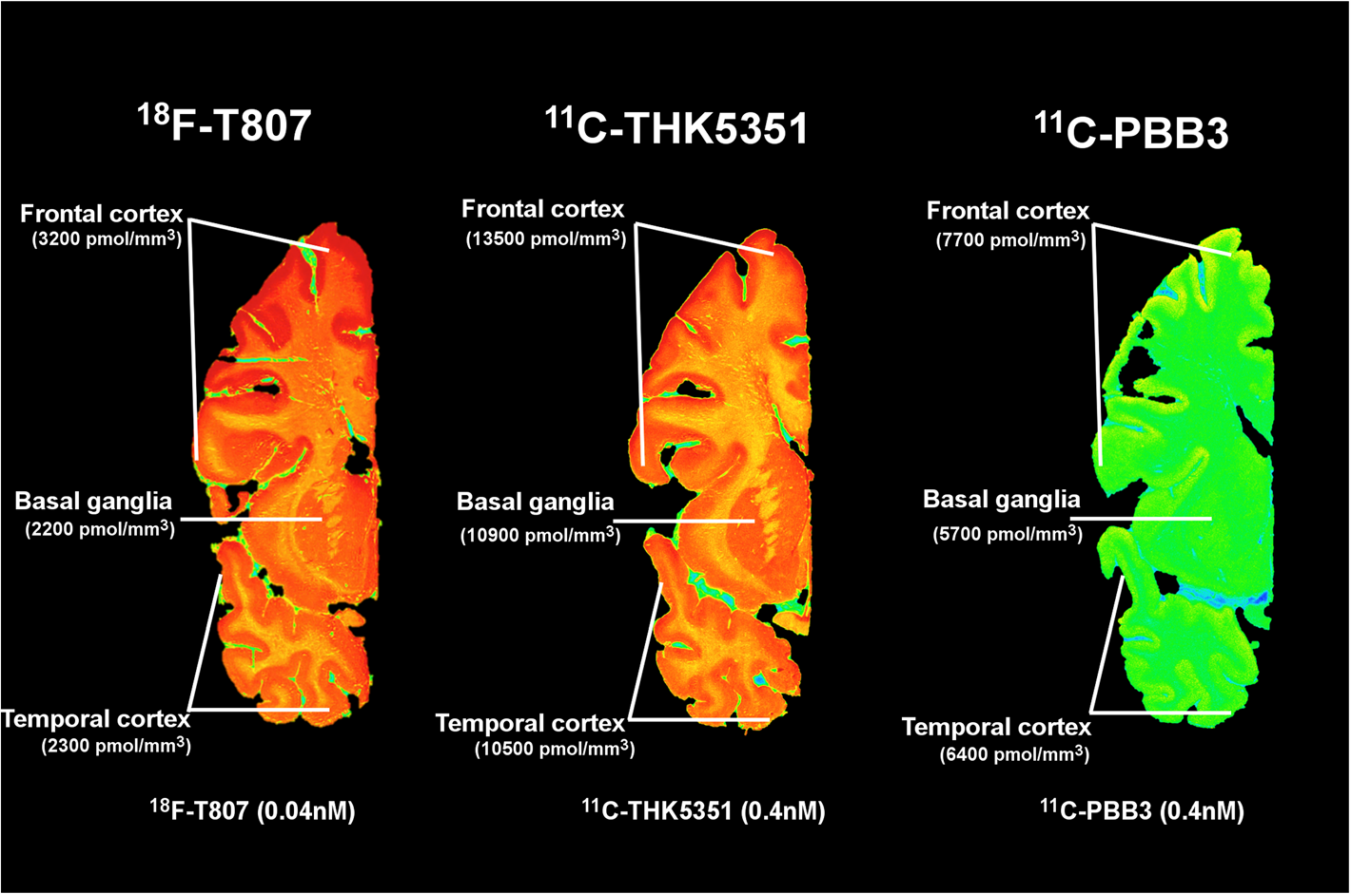
^18^F-T807, ^11^C-THK5351, and ^11^C-PBB3 autoradiography comparison of large frozen postmortem brain sections from AD3. The figure shows the total binding of ^18^F-T807, ^11^C-THK5351, and ^11^C-PBB3

Figure 5 shows the regional distribution of three of the tau PET tracers (T807, THK5351, and PBB3) in adjacent sections of AD3. ^18^F-T807 and ^11^C-THK5351 showed regional binding in both the frontal and temporal cortices. The regional ^11^C-PBB3 binding distribution in the cortical area was similar to that for ^18^F-T807 and ^11^C-THK5351, but less intense (Fig. 5). Semiquantitative analyses indicated that binding was twice as extensive for ^11^C-THK5351 as for ^11^C-PBB3. For example, in the frontal cortex, the binding was 13,500 pmol/mm^3^ for ^11^C-THK5351 and 7700 pmol/mm^3^ for ^11^C-PBB3 (*see* Additional file 1: Tables S1, S2, and S3 for all semiquantitative data). The same result was observed in the temporal cortex: 10,500 pmol/mm^3^ and 6400 pmol/mm^3^ for ^11^C-THK5351 and ^11^C-PBB3, respectively. Binding in subcortical areas and white matter was observed for both tracers. The semiquantitative data showed less binding for ^18^F-T807 than for ^11^C-THK5351 or ^11^C-PBB3, but the ^18^F-T807 concentration was ten times lower, so a direct comparison was not possible.

**Fig. 6:**
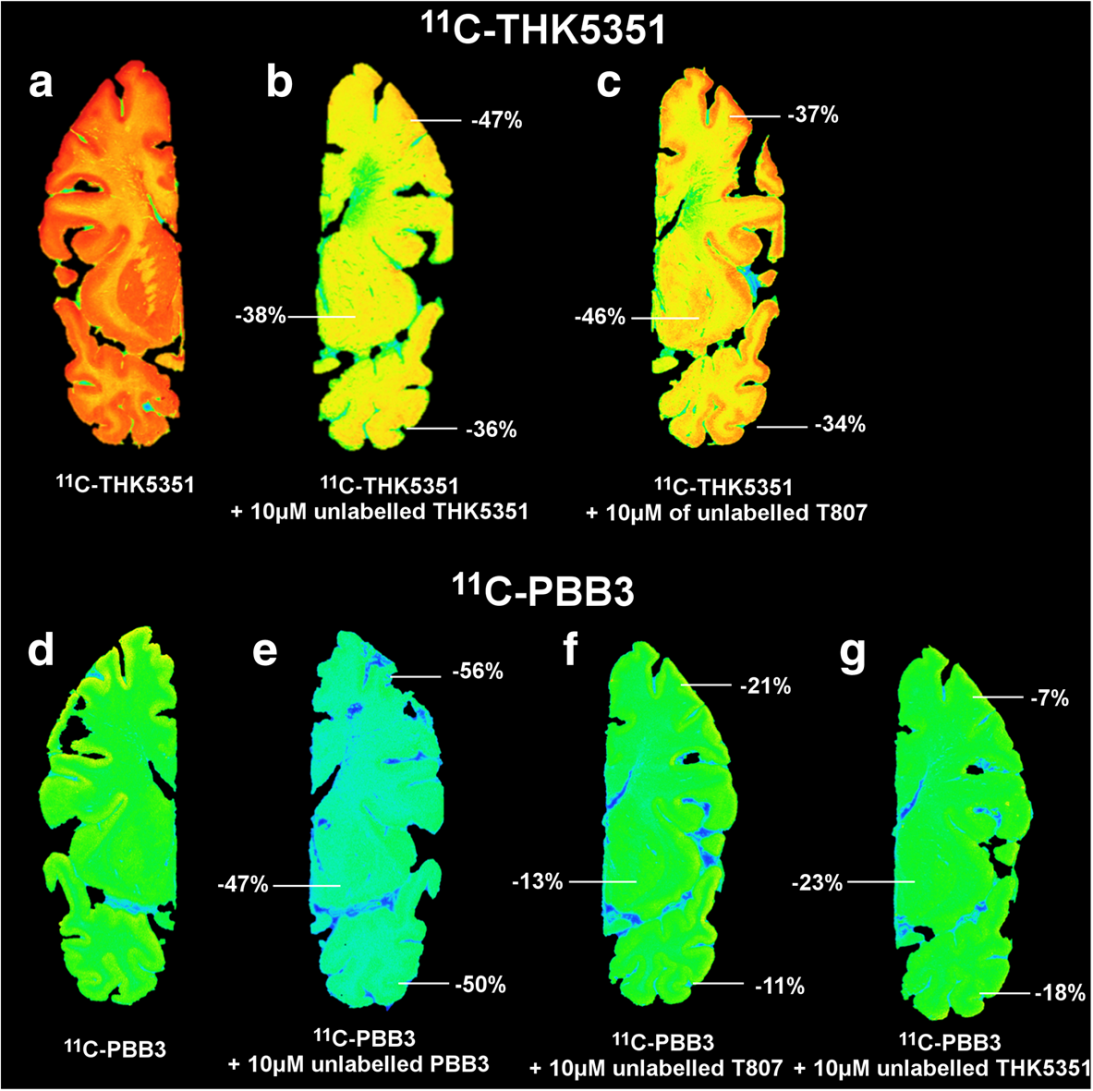
^11^C-THK5351 and ^11^C-PBB3 autoradiography competition with unlabeled THK5351, PBB3, and T807. The percentage inhibition of binding by the unlabeled compound in AD3 tissue for ^11^C-THK5351 and ^11^C-PBB3 autoradiography. **a**–**c** ^11^C-THK5351 autoradiography. **a** Total binding. **b** Competition with 10 μM unlabeled THK5351. **c** Competition with 10 μM unlabeled T807. **d**–**g** ^11^C-PBB3 autoradiography. **d** Total binding. **e** Competition with 10 μM unlabeled PBB3. **f** Competition with 10 μM unlabeled T807. **g** Competition with 10 μM unlabeled THK5351

Figure 6 shows a panel of autoradiography results for ^11^C-THK5351 and ^11^C-PBB3, with THK5351, PBB3, and T807 as unlabeled competitors in AD3 tissue. Visual assessment indicated that the regional distribution was similar throughout the whole cortical ribbon for the two ^11^C tracers (Fig. 6a, d). Qualitatively, ^11^C-PBB3 seems to be less extensively bound in white matter. The addition of unlabeled THK5351 (10 μM) displaced 47% of the ^11^C-THK5351 binding in the frontal gyrus and 36% in the temporal region (Fig. 6b). The addition of unlabeled T807 (10 μM) displaced almost 37% of the ^11^C-THK5351 binding in the frontal gyrus and 34% in the temporal region. In the subcortical region, putamen, and globus pallidus, T807 blocked 46% of ^11^C-THK5351 binding (Fig. 6c).

Similar autoradiography displacement experiments performed with ^11^C-PBB3 showed displacement of 56% with unlabeled PBB3 (10 μM) in the frontal gyrus and 50% in the temporal gyrus (Fig. 6e). The addition of unlabeled T807 (10 μM) led to 21% blocking in the frontal gyrus and 11% in the temporal gyrus (Fig. 6f). Addition of unlabeled THK5351 to ^11^C-PBB3 led to 7% inhibition of binding in the frontal cortex and 18% in the temporal cortex (Fig. 6g). The semiquantitative analyses are presented in Table 2, which shows the mean percentage inhibition of binding for ^11^C-PBB3 and ^11^C-THK5351 on addition of the unlabeled competitor in three AD tissue samples (*see* Additional file 1: Tables S1, S2, and S3 for the total binding, nonspecific binding, and specific binding values for each brain sample, respectively).

**Table 2:** Semiquantitative analysis of competition autoradiography using ^11^C-THK5351, ^11^C-PBB3, ^18^F-T807, and unlabeled THK5351, PBB3, and T807

	+ Unlabeled THK5351 (% inhibition)	+ Unlabeled PBB3 (% inhibition)	+ Unlabeled T807 (% inhibition)
^11^C-THK5351			
Frontal cortex	−52 ± 15	Experiment not performed	−27 ± 10
Insula	−43 ± 14		−27 ± 2
Temporal cortex	−41 ± 15		−29 ± 13
Basal ganglia	−49 ± 17		−42 ± 5
^11^C-PBB3			
Frontal cortex	−22 ± 5	−53 ± 3	−18 ± 3
Insula	−19 ± 1	−46 ± 2	−14 ± 6
Temporal cortex	−27 ± 9	−50 ± 9	−9 ± 4
Basal ganglia	−21 ± 3	−48 ± 1	−18 ± 4
^18^F-T807			
Frontal cortex	Experiment not performed	Experiment not performed	−35 ± 10
Insula			−27 ± 8
Temporal cortex			−30 ± 15
Basal ganglia			−26 ± 4

This table shows the extent of inhibition of binding (as a percentage) by unlabeled T807 of ^11^C-THK5351 by unlabeled THK5351 and T807; of ^11^C-PBB3 by unlabeled THK5351, PBB3, and T807; and of ^11^C-T807. The values presented are mean ± SD of values from three Alzheimer’s disease brain samples

### Competition binding study between ^3^H-deprenyl and unlabeled THK5117 or T807

**Fig. 7:**
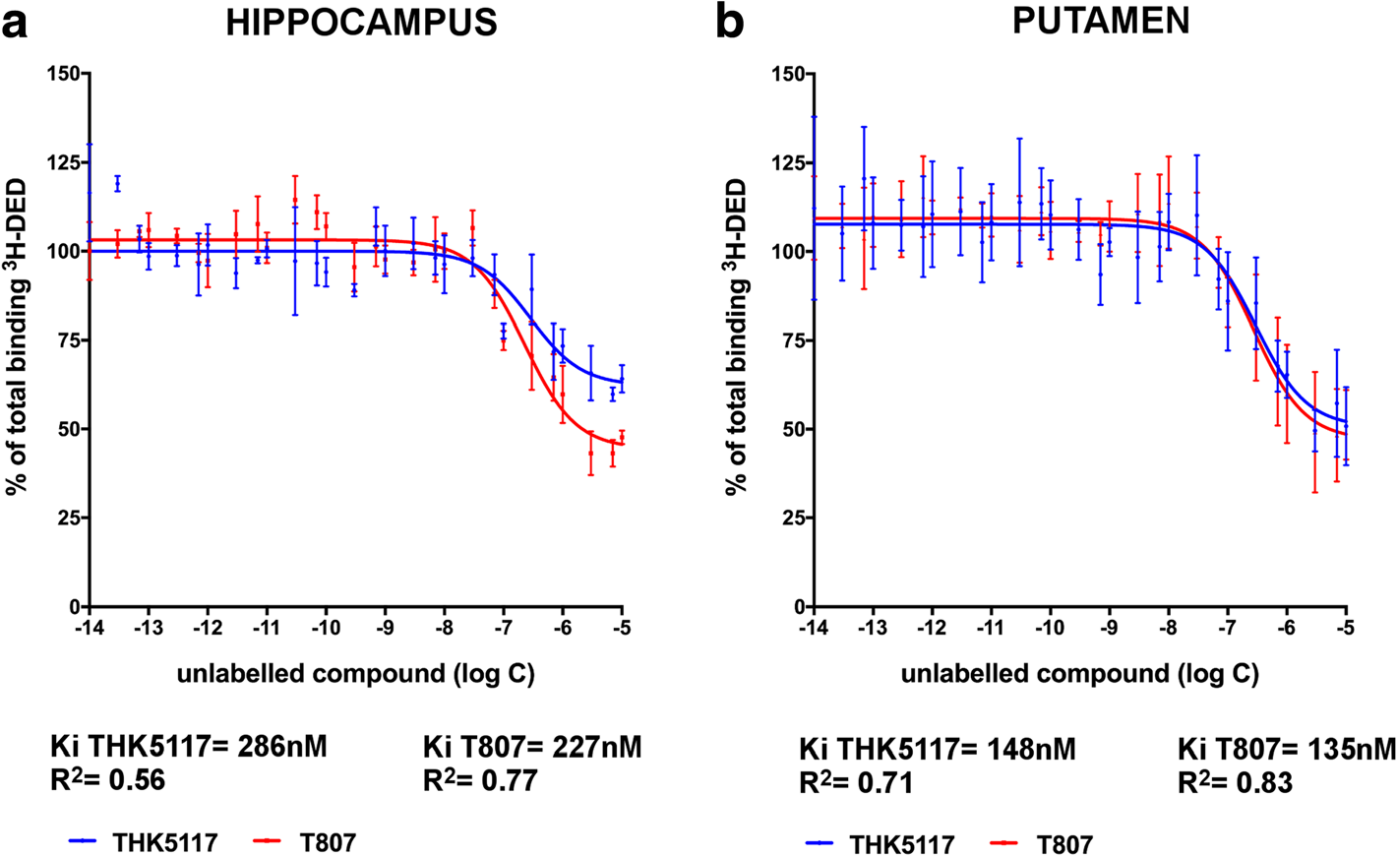
^3^H-Deprenyl (^3^H-DED) competition with unlabeled THK5351 and T807. Competition binding studies using increasing concentrations of unlabeled THK5117 or unlabeled T807 (1.10^−14^ to 1.10^−5^ M) with ^3^H-DED in hippocampus (**a**) and putamen (**b**) brain homogenates of two Alzheimer’s disease (AD) cases. Error bars represent the SEM of two experiments in triplicate for two AD cases. *K*_i_ Inhibition constant

The results of competition binding studies between ^3^H-deprenyl and unlabeled THK5117 or T807 in the hippocampus and putamen brain homogenates are presented in Fig. 7. Competition with ^3^H-deprenyl was observed in the hippocampus, with *K*_i_ values of 286 nM for THK5117 and 227 nM for T807. Similar results were obtained in the putamen, with *K*_i_ values of 148 nM for THK5117 and 135 nM for T807.

### Competition autoradiography binding study comparison between ^3^H-THK5351 and unlabeled THK5351, deprenyl, and T807

**Fig. 8:**
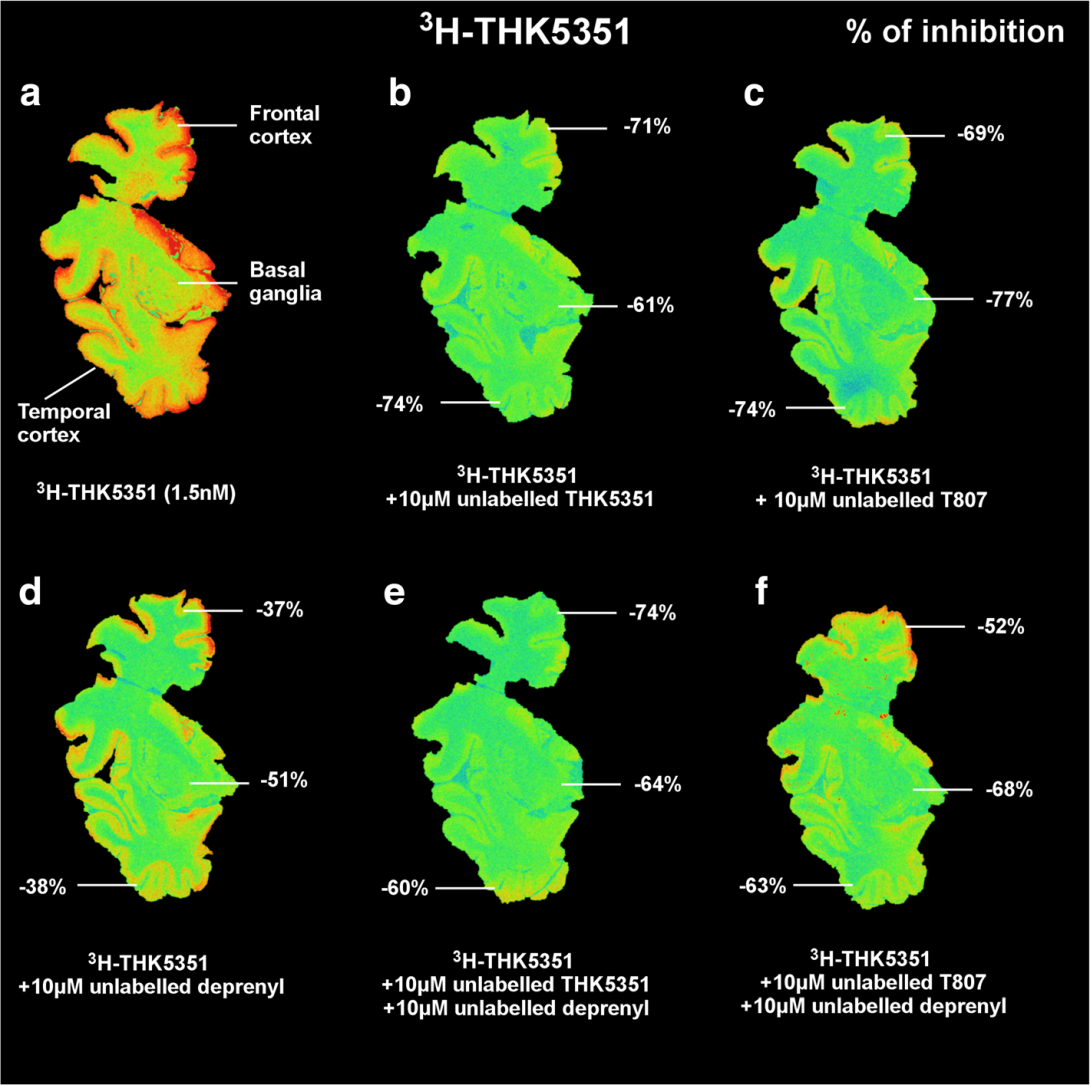
^3^H-THK5351 autoradiography competition with unlabeled THK5351, T807, and deprenyl. ^3^H-THK5351 comparisons in large frozen postmortem brain sections from a patient with Alzheimer’s disease (AD1). **a** Total binding (1.5 nM of ^3^H-THK5351). **b** Competition with 10 μM unlabeled THK5351. **c** Competition with 10 μM unlabeled T807. **d** Competition with 10 μM unlabeled deprenyl. **e** Competition with 10 μM unlabeled THK5351 + 10 μM unlabeled deprenyl. **f** Competition with 10 μM unlabeled T807 + 10 μM unlabeled deprenyl. The percentage inhibition of binding by the unlabeled compounds in AD1 tissue for the various regions is marked

Figure 8 shows the competition autoradiography results from large frozen brain sections, with ^3^H-THK5351 in competition with unlabeled THK5351, deprenyl, or T807, as well as combinations of the unlabeled compounds. In this set of experiments, unlabeled THK5351 displaced about 70% of the ^3^H-THK5351 binding in the frontal and temporal cortical areas and 61% in the basal ganglia (Fig. 8b). Unlabeled T807 displaced about 70–74% of ^3^H-THK5351 binding in the frontal and temporal cortices and up to 77% in the basal ganglia (Fig. 8c). Unlabeled deprenyl displaced more than 40% in both frontal and temporal cortices but more than 50% in the basal ganglia (Fig. 8d).

## Discussion

The aim of this study was to compare, using in vitro binding assays and in vitro autoradiography, the available tau PET tracers in the same postmortem tissue from patients with AD. The comparison of the two THK compounds, THK5117 and THK5351, is discussed first. THK5117 was developed as a tau PET ligand and was found to bind to white matter in the brain. THK5351, the latest THK compound, was developed as a pure *S*-form enantiomer to lower white matter binding [24]. The saturation binding curve of ^3^H-THK5351 showed two binding sites and good binding properties, with a *K*_d_ of 5 nM in the range of the optimum *K*_d_ for a PET tracer. These results are similar to those obtained with THK5117, which also had two binding sites in the saturation experiments [10]. The head-to-head direct comparison between the two THK compounds using single concentration binding assays in several brain regions showed similar distribution patterns in all the regions studied. These results confirm that the *S*-form is the only active form because we used half the concentration for ^3^H-THK5351 as we did for ^3^H-THK5117. Autoradiography with frozen hemispheric coronal sections showed similar distribution patterns throughout the cortical ribbon as well as a similar lamination binding pattern, indicating that the tracers target the same tau deposits. We observed more nonspecific binding with THK5117 than with THK5351.

In this study, the binding assay competition between ^3^H-THK5351 and unlabeled THK5117, unlabeled THK5351, and unlabeled T807 showed that the three unlabeled tracers compete with ^3^H-THK5351 for at least two binding sites. The THK compounds bind to two similar binding sites: one with a high affinity (*K*_i1_ 0.2 pM) and the other with a much lower affinity (*K*_i2_ approximately 20 nM). T807 seems to behave differently from ^3^H-THK5351. Indeed, it was clear that even though it competed for the same high-affinity binding site, T807 competed with fourfold less affinity than THK5351, in the nanomolar range (*K*_i2_ 76 nM). This suggests that targeting of the binding sites in the nanomolar range visible in PET studies will probably differentiate between T807 and the THK compounds. To our knowledge, this is the first study comparing the three families of tau PET tracers in the same brain tissue. The slightly different results observed between ^3^H and ^11^C autoradiography studies were most probably due to the emission type as well as to the special protocols needed for the different isotopes. All the autoradiography protocols have been optimized in-house in order to use the optimal binding condition. No ethanol was used in either the binding or rinsing buffer. Three previous studies have compared the different tau PET tracer families in two-by-two batches. The first compared ^11^C-PBB3 and ^18^F-AV1451 [25]; the second compared ^3^H-AV1451 and ^3^H-THK523 (one of the first-generation THK compounds) [26]; and the third compared ^18^F-T808 (in the same family as T807) and THK5105 (THK family compound) [27].

Autoradiography studies on frozen hemispheric brain sections using ^11^C-THK5351 are reported for the first time in this study, to our knowledge. Visual assessment showed similar regional binding distributions for ^18^F-T807, ^11^C-THK5351, and ^11^C-PBB3 in all the analyzed regions. However, using semiquantitative analyses, the specific binding values for ^11^C-PBB3 were lower than those for ^11^C-THK5351. This difference in binding intensity may be the result of the molecules targeting different subtypes of tau deposit, which express binding sites with different binding affinities. The semiquantitative data for ^18^F-T807 were also lower than for either ^11^C-THK5351 or ^11^C-PBB3, but the concentrations used for the autoradiography were ten times lower because of the high radioactivity of ^18^F.

The addition of unlabeled T807 decreased the ability to block ^11^C-PBB3 binding compared with ^11^C-THK5351 binding. Those results support the binding assay data. Indeed, the THK compound and T807 seem to share similar binding sites, although they do not have the same affinity for them. In the autoradiography studies, we also observed that unlabeled T807 displaced the binding of ^11^C-THK5351 in the subcortical regions slightly more than in the cortical region, confirming the off-target binding of T807 in the subcortical region.

Only a few studies are available to compare different tau tracer binding sites in the same populations. Declercq et al. recently compared THK5105 and T807 and suggested a possible similar binding site for the two tracers [27]. Cai et al. designed a study to develop a new tau deposit tracer with high affinity compared THK523 and T807 with PIB. Their study suggested that THK523 and T807 have two separate binding sites on the NFTs and that those binding sites differ from the thioflavin-T binding site targeted by amyloid PET tracers [26]. In our study, both ^11^C-THK5351 and ^18^F-T807 showed binding in the basal ganglia region. Both THK5351 and AV1451 in vivo PET have shown high binding in the basal ganglia, probably at least partly reflecting off-target binding to MAO-A and MAO-B [15]. Recently, Ng et al. [20] demonstrated a diminution of ^18^F-THK5351 binding in patients after treatment with selegiline (deprenyl) (protocol consisting of one baseline ^18^F-THK5351 scan, then 1 week later, a second ^18^F-THK5351 scan 1 h after 10-mg oral dose of selegiline). Owing to our observation of similar MAO-B components for both THK5351 and T807, it would be important to perform similar experiments with in vivo ^18^F-T807 PET scans following the pretreatment with selegiline. The in vitro ^3^H-deprenyl binding competition assay with unlabeled THK5117 and unlabeled T807 showed an affinity of approximately 150 nM for both in the putamen and greater than 200 nM in the hippocampus. For tau binding, the *K*_i_ values of THK5117 and T807 are, respectively, 20 nM and 78 nM toward ^3^H-THK5351. The results of the autoradiography competition studies with unlabeled T807, THK5351, and deprenyl (selegiline) showed that unlabeled T807 displaced ^3^H-THK5351 binding as much as unlabeled THK5351 in the frontal and temporal cortices, but more in the basal ganglia. These results confirm our hypothesis that THK5351 and T807 behave similarly in AD brain tissue. Unlabeled deprenyl alone displaced more binding in the basal ganglia, a region richer in MAO-B. PBB3 seems to bind differently and to have a unique regional distribution. Moreover, a recent study by Ono et al. [25], who compared T807 and PBB3 head to head, showed that the two compounds bound to different sites. Regarding the MAO-A, a previous study has shown interaction with MAO-A for T807 [22, 23]. Note that in the present study we assessed the MAO-A component only for ^3^H-THK5117 using clorgyline (MAO-A inhibitor) and found a *K*_i_ value of 273 nM for clorgyline toward ^3^H-THK5117 (*see* Additional file 2: Figure S2).

This study has several limitations. It is important to note the differences in techniques between in vitro autoradiography on large frozen tissue sections (80 μm thick) and binding assays in brain homogenates. Indeed, the binding assay in brain homogenates might make more binding sites accessible for binding in comparison to in vitro autoradiography. Moreover, we used different isotopes—^3^H, ^11^C, and ^18^F—with different energy and emission, which could also be a limitation of the study. However, we consider that those different techniques provide valuable complementary information for the characterization of the different tracers. This is important to understanding the different binding sites of the tau PET tracers.

In this study, we focused on the THK compounds as well as PBB3 and T807. Recently, some new tau PET tracer candidates have been reported both at international congresses and in the literature: ^18^F-MK6240; ^18^F-RO6958948; ^18^F-GTP1 (Genentech Tau Probe 1); ^18^F-PI2620; ^18^F-JNJ64349311; and new analogues of PBB3, ^18^F-AM-PBB3 and ^18^F-PM-PBB3 [22, 28–31]. It will be interesting in the future to perform head-to-head comparisons including these new tau PET tracers.

Interestingly, the cryo-electron microscopic structure of tau fibrils was recently reported by Fitzpatrick et al. [32]. This new knowledge will allow in silico computer modeling and determination of different binding sites on the tau fibrils, similar to what has already been done for the amyloid fibrils [33]. This will also help in the characterization and optimization of tau PET tracers using in silico modeling and radiochemistry. Even though we still have to keep in mind that in silico and in vitro behavior is not the same as in vivo, the complementarity of all the different techniques is important for the characterization of PET tracers.

## Conclusions

To our knowledge, this is the first study to compare PBB3, T807, and THK5351 in the same AD brain tissue samples using both brain homogenate and autoradiography studies. The head-to-head comparison suggests that the THK compound and T807 target similar binding sites with different affinities but that PBB3 seems to target its own site. Both THK5351 and T807 show off-target binding to MAO-B with affinity similar to that of deprenyl. It is important to carry out more in vitro characterization of these compounds before proceeding to clinical studies. Indeed, the different PET tracers seem to bind to different subtypes of tau deposit; thus, knowing exactly what kind of tau deposit is being targeted could help with the diagnosis and add information about the neuropathological sequence.

## Additional files

Additional file 1: Table S1. Semi-quantitative analysis of ^11^C-THK5351 autoradiography in competition with unlabelled THK5351 and unlabelled T807. Unlabelled PBB3 was not studied. Specific binding was calculated as total binding minus non-specific (NSP) binding. **Table S2.** Semi-quantitative analysis of ^11^C-PBB3 autoradiography in competition with unlabelled THK5351, unlabelled T807 and unlabelled PBB3. Specific binding was calculated as total binding minus non-specific (NSP) binding. **Table S3.** Semi-quantitative analysis of ^18^F-T807 autoradiography in competition with unlabelled T807. Neither unlabelled THK5351 nor unlabelled PBB3 were studied. Specific binding was calculated as total binding minus non-specific (NSP) binding. (DOCX 109 kb)

Additional file 2: Figure S1. Competition binding assay with H-THK5117 and unlabelled clorgyline. Competition binding studies using ^3^H-THK5117 (3nM) binding in hippocampus brain homogenate from one AD cases using increasing concentration of clorgyline (10^-14^ -10^-5^). Errors bars represent the standard errors of the mean from three experiments in triplicate. (DOCX 229 kb)

## Abbreviations

AD: Alzheimer’s disease
BSA: Bovine serum albumin
^11^C-THK5351: (*S*)-1-fluoro-3- (2-(6-([^11^C]methylamino) pyridin-3-yl) quinolin-6-yloxy) propan-2-ol
FC: Frontal cortex
FDDNP: 2-(1-{6-[(2-[fluorine-18]fluoroethyl)(methyl)amino]-2-naphthyl}-ethylidene)malononitrile
^3^H-THK5117: 1-Fluoro-3-((2-(4-([^3^H]methylamino)phenyl)quinolin-6-yl)oxy)propan-2-ol
Hip: Hippocampus
*K*_d_: Dissociation constant
*K*_i_: Inhibition constant
MAO: Monoamine oxidase
NFT: Neurofibrillary tangle
PET: Positron emission tomography
PHF-tau: Paired helical filaments
PIB: ^11^C-Pittsburgh compound B
PSL: Photostimulated luminescence
TC: Temporal cortex
