# Supplementary material for: Comparative binding properties of the tau PET tracers THK5117, THK5351, PBB3, and T807 in postmortem Alzheimer brains
Source: Alzheimers Res Ther. 2017 Dec 11;9:96. doi: 10.1186/s13195-017-0325-z (PMC5725799; doi:10.1186/s13195-017-0325-z)
Supplement: Supplementary file 1 — Semi-quantitative analysis of 11C-THK5351 autoradiography in competition with unlabelled THK5351 and unlabelled T807. Unlabelled PBB3 was not studied. Specific binding was calculated as total binding minus non-specific (NSP) binding. Table S2. Semi-quantitative analysis of 11C-PBB3 autoradiography in competition with unlabelled THK5351, unlabelled T807 and unlabelled PBB3. Specific binding was calculated as total binding minus non-specific (NSP) binding. Table S3. Semi-quantitative analysis of 18F-T807 autoradiography in competition with unlabelled T807. Neither unlabelled THK5351 nor unlabelled PBB3 were studied. Specific binding was calculated as total binding minus non-specific (NSP) binding. (DOCX 109 kb) [file 13195_2017_325_MOESM1_ESM.docx]

| ^11^C-THK5351 |  |  |  | Unlabelled THK5351 | | | |  | Unlabelled T807 | | | |  | Unlabelled PBB3 | | | |
| --- | --- | --- | --- | --- | --- | --- | --- | --- | --- | --- | --- | --- | --- | --- | --- | --- | --- |
|  |  |  |  | Total Binding | NSP Binding | Specific Binding | % of specific binding |  | Total Binding | NSP Binding | Specific Binding | % of specific binding |  | Total Binding | NSP Binding | Specific Binding | % of specific binding |
|  | AD1 | Frontal cortex |  | 14837 | 8920 | 5917 | 40 |  | 20368 | 15579 | 4789 | 24 |  |  | | | |
|  |  | Insula |  | 12461 | 8376 | 4085 | 33 |  | 17677 | 13255 | 4421 | 25 |  |  |  |  |  |
|  |  | Temporal cortex |  | 11587 | 8202 | 3386 | 29 |  | 16990 | 14662 | 2328 | 14 |  |  |  |  |  |
|  |  | Basal ganglia |  | 12694 | 7577 | 5118 | 40 |  | 18444 | 10866 | 7578 | 41 |  |  |  |  |  |
|  |  |  |  |  |  |  |  |  |  |  |  |  |  |  |  |  |  |
|  | AD2 | Frontal cortex |  | 22136 | 6755 | 15381 | 69 |  | 18167 | 14824 | 3344 | 18 |  |  |  |  |  |
|  |  | Insula |  | 16834 | 6886 | 9948 | 59 |  | 18439 | 13363 | 5076 | 28 |  |  |  |  |  |
|  |  | Temporal cortex |  | 16110 | 6673 | 9437 | 59 |  | 22271 | 13589 | 8683 | 39 |  |  |  |  |  |
|  |  | Basal ganglia |  | 19956 | 6390 | 13566 | 68 |  | 19008 | 11830 | 7177 | 38 |  |  |  |  |  |
|  |  |  |  |  |  |  |  |  |  |  |  |  |  |  |  |  |  |
|  | AD3 | Frontal cortex |  | 13509 | 7079 | 6431 | 48 |  | 23751 | 14792 | 8959 | 38 |  |  |  |  |  |
|  |  | Insula |  | 10875 | 6903 | 3972 | 37 |  | 19021 | 13574 | 5447 | 29 |  |  |  |  |  |
|  |  | Temporal cortex |  | 10529 | 6717 | 3813 | 36 |  | 19153 | 12673 | 6479 | 34 |  |  |  |  |  |
|  |  | Basal ganglia |  | 10939 | 6733 | 4207 | 38 |  | 20693 | 10872 | 9821 | 47 |  |  |  |  |  |

Supplementary table 1:

Supplementary table 1: Semi-quantitative analysis of ^11^C-THK5351 autoradiography in competition with unlabelled THK5351 and unlabelled T807. Unlabelled PBB3 was not studied. Specific binding was calculated as total binding minus non-specific (NSP) binding.

Supplementary table 2:

| ^11^C-PBB3 |  |  |  | Unlabelled THK5351 | | | |  | Unlabelled T807 | | | |  | Unlabelled PBB3 | | | |
| --- | --- | --- | --- | --- | --- | --- | --- | --- | --- | --- | --- | --- | --- | --- | --- | --- | --- |
|  |  |  |  | Total Binding | NSP Binding | Specific Binding | % of specific binding |  | Total Binding | NSP Binding | Specific Binding | % of specific binding |  | Total Binding | NSP Binding | Specific Binding | % of specific binding |
|  | AD1 | Frontal cortex |  | 2018 | 1579 | 439 | 22 |  | 4701 | 3919 | 782 | 17 |  | 6750 | 3317 | 3433 | 51 |
|  |  | Insula |  |  |  |  |  |  | 4409 | 3842 | 566 | 13 |  | 6043 | 3353 | 2690 | 45 |
|  |  | Temporal cortex |  | 2164 | 1569 | 596 | 28 |  | 4187 | 4009 | 178 | 4 |  | 5777 | 3425 | 2352 | 41 |
|  |  | Basal ganglia |  |  |  |  |  |  | 4004 | 3201 | 802 | 20 |  | 5645 | 2849 | 2796 | 50 |
|  |  |  |  |  |  |  |  |  |  |  |  |  |  |  |  |  |  |
|  | AD2 | Frontal cortex |  | 1960 | 1619 | 341 | 17 |  | 3847 | 3252 | 595 | 15 |  | 6238 | 3051 | 3187 | 51 |
|  |  | Insula |  | 1979 | 1580 | 400 | 20 |  | 3585 | 2838 | 748 | 21 |  | 5927 | 3100 | 2827 | 48 |
|  |  | Temporal cortex |  | 2336 | 1506 | 831 | 36 |  | 3874 | 3425 | 448 | 12 |  | 7210 | 2988 | 4222 | 59 |
|  |  | Basal ganglia |  | 1884 | 1517 | 367 | 19 |  | 3541 | 2790 | 751 | 21 |  | 5752 | 3052 | 2700 | 47 |
|  |  |  |  |  |  |  |  |  |  |  |  |  |  |  |  |  |  |
|  | AD3 | Frontal cortex |  | 2511 | 1832 | 678 | 27 |  | 4125 | 3247 | 879 | 21 |  | 7171 | 3132 | 4039 | 56 |
|  |  | Insula |  | 2220 | 1815 | 405 | 18 |  | 3555 | 3261 | 294 | 8 |  | 6249 | 3401 | 2848 | 46 |
|  |  | Temporal cortex |  | 2215 | 1818 | 397 | 18 |  | 3612 | 3217 | 396 | 11 |  | 6409 | 3203 | 3207 | 50 |
|  |  | Basal ganglia |  | 2058 | 1577 | 481 | 23 |  | 3143 | 2721 | 422 | 13 |  | 5775 | 2989 | 2786 | 48 |

Supplementary table 2: Semi-quantitative analysis of ^11^C-PBB3 autoradiography in competition with unlabelled THK5351, unlabelled T807 and unlabelled PBB3. Specific binding was calculated as total binding minus non-specific (NSP) binding.

Supplementary table 3:

| ^18^F-T807 |  |  |  | Unlabelled THK5351 | | | |  | Unlabelled T807 | | | |  | Unlabelled PBB3 | | | |
| --- | --- | --- | --- | --- | --- | --- | --- | --- | --- | --- | --- | --- | --- | --- | --- | --- | --- |
|  |  |  |  | Total Binding | NSP Binding | Specific Binding | % of specific binding |  | Total Binding | NSP Binding | Specific Binding | % of specific binding |  | Total Binding | NSP Binding | Specific Binding | % of specific binding |
|  | AD1 | Frontal cortex |  |  | | | |  | 3416 | 2028 | 1388 | 41 |  |  | | | |
|  |  | Insula |  |  |  |  |  |  | 2744 | 1851 | 893 | 33 |  |  |  |  |  |
|  |  | Temporal cortex |  |  |  |  |  |  | 2802 | 2018 | 784 | 28 |  |  |  |  |  |
|  |  | Basal ganglia |  |  |  |  |  |  | 2495 | 1895 | 599 | 24 |  |  |  |  |  |
|  |  |  |  |  |  |  |  |  |  |  |  |  |  |  |  |  |  |
|  | AD2 | Frontal cortex |  |  |  |  |  |  | 2488 | 1901 | 586 | 24 |  |  |  |  |  |
|  |  | Insula |  |  |  |  |  |  | 2469 |  |  |  |  |  |  |  |  |
|  |  | Temporal cortex |  |  |  |  |  |  | 3327 | 1801 | 1526 | 46 |  |  |  |  |  |
|  |  | Basal ganglia |  |  |  |  |  |  | 2280 | 1713 | 567 | 25 |  |  |  |  |  |
|  |  |  |  |  |  |  |  |  |  |  |  |  |  |  |  |  |  |
|  | AD3 | Frontal cortex |  |  |  |  |  |  | 3212 | 1916 | 1296 | 40 |  |  |  |  |  |
|  |  | Insula |  |  |  |  |  |  | 2346 | 1836 | 509 | 22 |  |  |  |  |  |
|  |  | Temporal cortex |  |  |  |  |  |  | 2349 | 1975 | 374 | 16 |  |  |  |  |  |
|  |  | Basal ganglia |  |  |  |  |  |  | 2372 | 1639 | 733 | 31 |  |  |  |  |  |

Supplementary table 3: Semi-quantitative analysis of ^18^F-T807 autoradiography in competition with unlabelled T807. Neither unlabelled THK5351 nor unlabelled PBB3 were studied. Specific binding was calculated as total binding minus non-specific (NSP) binding.
