# Supplementary material for: Comparative binding properties of the tau PET tracers THK5117, THK5351, PBB3, and T807 in postmortem Alzheimer brains
Source: Alzheimers Res Ther. 2017 Dec 11;9:96. doi: 10.1186/s13195-017-0325-z (PMC5725799; doi:10.1186/s13195-017-0325-z)
Supplement: Supplementary file 2 — Competition binding assay with H-THK5117 and unlabelled clorgyline. Competition binding studies using 3H-THK5117 (3nM) binding in hippocampus brain homogenate from one AD cases using increasing concentration of clorgyline (10-14 -10-5). Errors bars represent the standard errors of the mean from three experiments in triplicate. (DOCX 229 kb) [file 13195_2017_325_MOESM2_ESM.docx]

Supplementary figure 1:

Methods:

Competition binding assay using hippocampus brain homogenate of one AD case was performed using ^3^H-THK5117 (3nM in PBS+0.1%BSA pH7.4) and increasing concentration of clorgyline (10^-14^-10^-5^M), MAO-A inhibitor. After 2 hours' incubation at room temperature, the binding assay was terminated by filtration through glass fibre filters presoaked for at least 3 hours in 0.3% polyethylenimine. To do so, the filters were rinsed and filtered three times using cold binding buffer and then, the radiation on the filter was then quantified using a Beckman scintillation counter. The data from all the binding studies were analyzed using graphpad prism 7 software.

Results:

Competition studies in the hippocampus between unlabelled clorgyline and ^3^H-THK5117 showed a Ki of 273 nM.


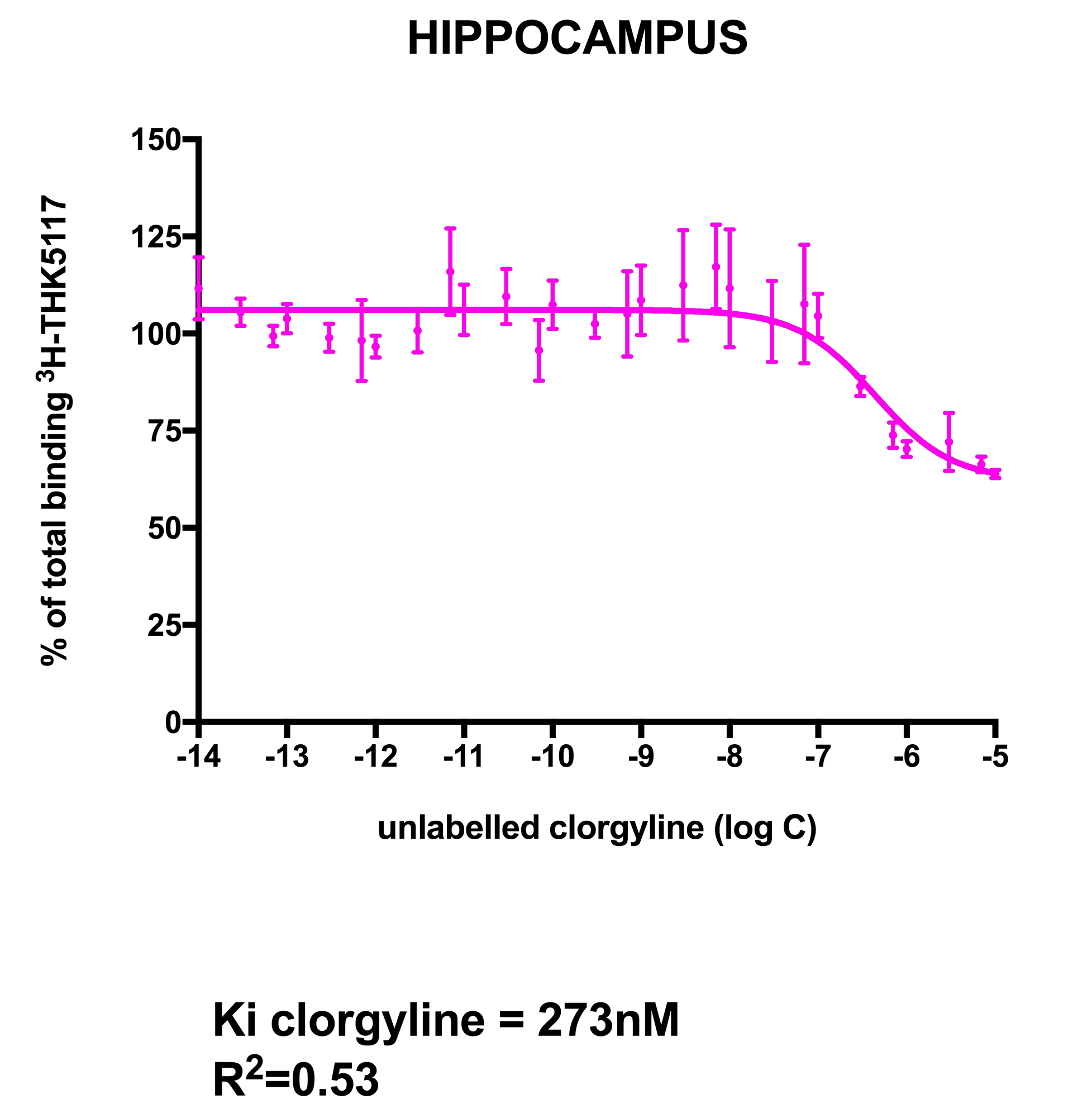


**Supplementary figure 1: Competition binding assay with 3H-THK5117 and unlabelled clorgyline.**

Competition binding studies using ^3^H-THK5117 (3nM) binding in hippocampus brain homogenate from one AD cases using increasing concentration of clorgyline (10^-14^-10^-5^). Errors bars represent the standard errors of the mean from three experiments in triplicate.
